# Coating with Autologous Plasma Improves Biocompatibility of Mesh Grafts *In Vitro*: Development Stage of a Surgical Innovation

**DOI:** 10.1155/2013/536814

**Published:** 2013-09-17

**Authors:** Holger Gerullis, Evangelos Georgas, Christoph Eimer, Christian Arndt, Dimitri Barski, Bernhard Lammers, Bernd Klosterhalfen, Mihaly Borós, Thomas Otto

**Affiliations:** ^1^Department of Urology, Lukas Hospital, Preußenstraße 84, 41464 Neuss, Germany; ^2^West German Cancer Center (WTZ), University of Essen, Essen, Germany; ^3^German Centre for Assessment and Evaluation of Innovative Techniques in Medicine (DZITM), Germany; ^4^Department of Surgery, Lukas Hospital, Neuss, Germany; ^5^German Centre for Implant-Pathology, Düren, Germany; ^6^Department of Experimental Surgery, University of Szeged, Hungary

## Abstract

*Purpose*. To investigate mesh coating modalities with autologous blood components in a recently developed *in vitro* test system for biocompatibility assessment of alloplastic materials. *Materials and Methods*. Seven different mesh types, currently used in various indications, were randomly investigated. Meshes were coated prior to cultivation with autologous peripheral blood mononuclear cells (PBMCs), platelets, and blood plasma. Pretreated meshes were incubated over 6 weeks in a minced tissue assay, representative for fibroblasts, muscle cells, and endothelial cells originating from 10 different patients. Adherence of those tissues on the meshes was microscopically investigated and semiquantitatively assessed using a previously described scoring system. *Results*. Coating with peripheral blood mononuclear cells did not affect the adherence score, whereas coating with platelets and blood plasma increased the score suggesting improved biocompatibility *in vitro*. The previous ranking of native meshes remained consistent after coating. *Conclusion*. Plasma coating of meshes improves their biocompatibility score in a novel *in vitro* test system.

## 1. Introduction

The use of alloplastic materials is widely spread in surgery for hernia, incontinence, and prolapse. The assessment of meshes prior to their clinical use remains reasonable to minimize complications. There are several models for assessing different meshes with regard to their biomechanic characteristics [1, 2]. Quality control for surgical meshes is an important issue. It is likely to get increasingly important in the future considering the ongoing discussion about new regulations for the approval of medical devices as well as intense pre- and postmarket surveillance [3]. 

Currently, predictive information on the biocompatibility and side effect probability is rare. Klinge and Klosterhalfen presented a very valuable classification of surgical meshes for hernia repair based on the analyses of 1,000 explanted meshes. [4]. The results of a recent *in vitro* approach [5] have been successfully validated in an animal long-term study following standardized recommendations for the assessment of surgical material and methods, IDEAL (Innovation, Development, Exploration, Assessment, and Long-term study) [6, 7]. This method warrants further development and evaluation as a possible manufacturer-independent tool for pre- and postmarket evaluation of meshes. 

In current understanding, an optimal surgical mesh permits the transmigration and localisation of host cells and inhibits the adherence of visceral organs in order to avoid arrosion, foreign body induced pain, and so forth. Preoperative coating of meshes, with a protective layer on the visceral side of the mesh, has been previously investigated, mostly in *in vivo* approaches. These meshes reduced a foreign body reaction and improved biocompatibility. There are now, in fact, clear indications that surface modifications of meshes can help to influence a tissue reaction *in vivo* [8, 9]. However, *in vitro* approaches for native and modified mesh assessment are still scarce. The objective of this study was, therefore, to investigate the effect of coating of surgical meshes with autologous blood components using a recently established *in vitro* model for biocompatibility assessment.

## 2. Material and Methods 

### 2.1. General

The method has been reported previously and represents a semiquantitative approach, measuring the adherence of different tissues on the meshes' surface using a modification of the approach initially described by Melman and coworkers [5, 10]. 

### 2.2. Meshes/Patients

Seven meshes currently used for various indications such as hernia repair, pelvic organ prolapse (POP), and stress urinary incontinence (SUI) were studied.  Table 1 provides a short overview on important material characteristics. After receiving informed consent, we harvested tissue probes of muscle, fascia, and renal vein from 10 patients undergoing right side nephrectomy. Tissue processing was identical in all patients. All patients provided blood samples for further processing and isolation of blood components for subsequent mesh coating. Each mesh was tested with tissue and cells of each patient for comparison.

### 2.3. Blood Sample Processing

Peripheral blood mononuclear cells (PBMCs), platelets, and plasma were used. Separation of PBMCs was accomplished through density gradient centrifugation using Ficoll [11]. For isolation of platelets and respective mediators the advanced tissue regeneration system (ATR by Curasan Inc.) was used (http://www.curasan.de/de/produkte/dental/atr/atr.php) [12]. Plasma preparation followed the classical method of Crowley [13]. 

### 2.4. Mesh Coating

**Figure 1:**
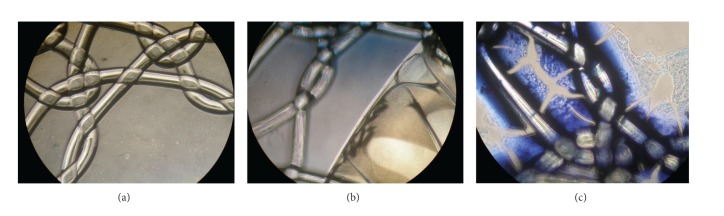
Plasma coating. The figure shows PVDF: (a) native, (b) after 12 hrs plasma incubation, and (c) after 12 hrs plasma incubation and trypan blue staining. In (b) and (c) plasma is adherent to mesh filaments whereas noncovered parts of the mesh appear native as in (a).

After isolating the three different blood components of each patient, we incubated the meshes (2 × 2 cm) with 10 mL of the respective suspension and incubated them over 12 hrs prior to testing with tissue. Successful plasma coating is exemplarily shown in Figure 1.

### 2.5. Tissue Preparation

The investigation of the adherence of specific cells has been previously described [14]. Our initial results with cell culture did not reveal sufficient cell growth; thus, we decided to use a minced tissue culture approach for the following investigations. Coincubation of implants with tissue clusters was also supported by the idea of reduced artificial modification processes due to shorter culture processing. In detail we proceeded as follows [5]: we extracted tissue probes originating from muscle, fascia, and renal vein at a length of 0.5–1.0 cm. Probes were incubated with Liforlab at room temperature. After crushing, we incubated the tissue with PBS and after 2 additional washing procedures we incubated DMEM/F12 plus 10% serum and 1%-glutamine, +1%Pen/Strep. Tissue probes were transferred to the incubator. After successful expanding and growing (80–90% adherent growth) of tissue pellets, the different alloplastic materials which previously had been prepared in 2 × 2 cm fragments were added. Thus, the prepared and expanded tissue probes consisting of myoblasts, endothelial cells, and fibroblasts which reflect the relevant tissues of the pelvic floor were used. Immunocytochemistry with specific cell markers was performed. The presence of myoblasts was confirmed by *α*-sarcomeric actin and desmin as markers of myogenic differentiation. Fibroblasts were stained with antibodies targeting vimentin, whereas antibodies against CD34 were used for endothelial cells. Data were generated for tissue samples of patients.

### 2.6. Morphological Study

The tissue cultures were maintained up to 4 months with frequent changes of medium, and assessment was repeated if possible. Meshes were investigated microscopically with regard to interstructural tissue connections and quantity of mesh adherent cells. The semiquantitative assessment scheme was based on the maximum number of adherent cells and size of tissue clusters per vision field. The adherence on the meshes was ranked none, fair, good, and excellent [10]. The respective scores of coated versus uncoated meshes were compared for each patient and each coating separately.

## 3. Results

Tissue growth was comparable in all approaches over 6 weeks. We did not observe macroscopic differences in the gross appearance of the meshes after tissue culture. No signs of infection were observed. 

### 3.1. Microscopic Results

**Figure 2:**
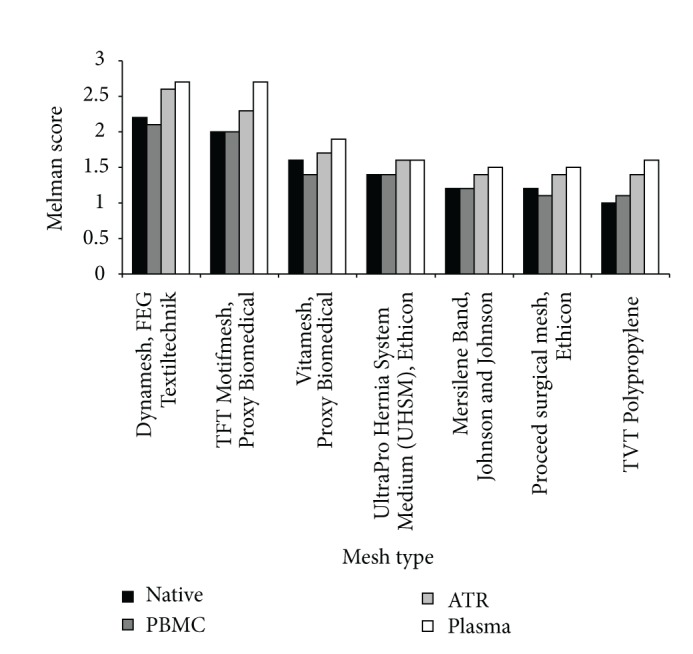
Mesh ranking. Frequency distribution value (*y*-axis) of the modified Melman score for each mesh with the respective coatings is compared among the investigated 7 meshes.

The testing of the biocompatibility of myoblasts, endothelial cells, and fibroblasts was observed under addition of BioGlue. According to the descriptive/semiquantitative approach described above, we revealed a ranking of the native meshes after 6 weeks [5]. The modified Melman score was subsequently used to the three different coating approaches for each patient. We observed comparable tissue ingrowth to the native mesh when analysing the PBMC-incubated meshes. Interestingly, the meshes previously incubated with ATR (Curasan Inc.) and the plasma coated meshes revealed a slightly better performance.  Table 2 and Figure 2 show the ranking of the investigated native meshes and the different coating modifications. This trend was reproduced after 4 months of tissue culture. All individuals revealed comparable effects of tissue ingrowth in the native state and after coating with different blood components.

## 4. Discussion

Many scientists agree that the choice of the appropriate mesh is as important as the surgical technique when determining clinical outcomes after mesh applying surgeries independent of the particular indication [15, 16]. Currently, a plethora of commercially available meshes makes the decision of which mesh to apply very difficult. Two FDA warnings from 2008 and 2011 reported more than 3.500 severe adverse events after mesh application, mostly in POP and SIU patients. As a consequence the FDA recommended consideration of regulatory changes including an upgrading in risk classifications for meshes, clinical studies to address the risks and benefits of meshes, and expanded postmarket monitoring of device performance [17]. It was suggested that data on adequate functional performance and material safety should get increasingly in the focus of premarket review for mesh devices. Thus, preclinical investigations in terms of bench and/or animal testing could represent a new standard requirement to confirm that engineering specifications are met and that the material and/or specific modification chosen for a mesh is sufficiently biocompatible. To date, there are numerous reports of mesh modification approaches in order to improve their biocompatibility. The permanent character of a foreign body implant may cause persistent and increased inflammation with ongoing collagen disposition leading to extensive fibrosis. Impaired host acceptance of the implanted mesh is likely to appear through chronic inflammation and extensive fibrosis [18]. In order to tackle the problem of extensive foreign body reaction (FBR) initiated by early local inflammation, several researchers have modified the chemical and physical properties of meshes by different coating approaches resulting on altered local reaction and tissue response, mostly using *in vivo* experiments. Various compounds have been tested so far for mesh coating purposes, however, the majority in *in vivo* models, mostly after setting a pathological defect being repaired by the investigated meshes [9, 19–22]. Besides numerous *in vivo* experiments, Bryan and coworkers provide an *in vitro* model to facilitate mesh choice in uncomplicated hernia repair by quantitatively determining of neutrophil activation and degranulation in different mesh types [23]. Their approach represents one of the few *in vitro* assessment tools for meshes, currently available in the literature. In their experiments, reactive oxygen species (ROS), released by activated neutrophils leading to nonspecific host tissue damage and potential mechanical weakening, have been measured on the surface of 6 different meshes. The authors investigated native, nonmodified meshes. However, they concluded mesh structure being a greater determinant of ROS release than chemical composition. It seems likely that their sophisticated assay could be used for mesh assessment after different coating approaches as well. This would be a conclusive further development comparable to the approach presented here, which represents an advancement of the initially described *in vitro* assessment tool for native meshes [5]. The aim of this study was to implement and assess an easy mesh-coating procedure *in vitro* and to investigate if coating of meshes with autologous blood components shows different *in vitro* interaction characteristics with different tissues types compared to native meshes. We used autologous blood components as they are relatively easy to obtain from the respective patients and contain relevant cells and substances involved in humoral immune defence. This approach was based on the assumption that the extent to which an implanted alloplastic material elicit an acute local inflammatory response has impact on the long-term outcome when applied *in vivo* [24]. In order to investigate cellular and noncellular components, we separately investigated PBMC, plasma, and platelets with the respective mediators. Incubation with peripheral blood mononuclear cells did not result in modification of the adherence score for the investigated tissues. This may be explained by the reduced ability of those cells to maintain in permanent contact with the polymer surface of the meshes as previously shown [5]. In contrast, blood plasma and ATR resulted in better adherence performance and increased biocompatibility in all meshes. A main limitation of this study is that no inflammatory reaction as normally cascading *in vivo* was imitated as the *in vitro* approach was sterile. However, predictability for *in vivo* circumstances is currently being tested in an animal model in sheep for the coated and noncoated meshes. First results describe a predictive value of the *in vitro* system for mesh performance in *in vivo* surroundings [6].

An interesting observation in the current study is that all meshes which have previously been ranked with regard to their biocompatibility performance show an increased score after plasma coating and maintain their position in the ranking, compared to the other investigated meshes. This supports the thesis that coating with plasma may have an effect independent of the mesh; however, at least *in vitro*, all meshes could improve their performance but low ranked meshes could not increase their position compared to natively better positioned counterparts. The thesis of Bryan and coworkers can thereby be supported: mesh structure seems to be an important determinant of the *in vitro* performance in the native and coated configuration of a mesh [23]. Mesh related complications are known to be related to extensive local inflammation, representing the first step of a foreign body reaction [25]. This foreign body reaction after implantation of a mesh is assumed to be triggered by secretion of a variety of proteins attracting inflammatory cells to migrate to the site of injury, finally leading to extracellular matrix regulation and collagen deposition [26]. In a recent study, Brandt and colleagues investigated the effect of mesh coating (PVDF, polyvinylidene fluoride) with different substances affecting the cortisone metabolism. In their *in vivo* approach they found that hydrocortisone and spironolactone protected from inflammatory response ended up in smaller granuloma at the implant site of the mesh and decreased the collagen formation [27]. Their approach suggested that the respective coating approaches are a possible way to attenuate local inflammatory processes in order to reduce FBR. This is supported by other research groups who show altered local cell activation and tissue responses after modifying the chemical and/or physical properties of meshes via coating attempts, thus, leading to the hypothesis that coating of polymer surfaces may represent an opportunity to improve mesh integration and biocompatibility [28]. Assuming the adherence performance of tissue on a mesh as possible marker for its biocompatibility seems logic independent from the respective clinical use of the implant. Although, an exaggerated foreign body reaction/tissue response is discussed to be related to clinical complications, a positive role in mesh incorporation at the implant site may be triggered by bioactive mediators like epidermal growth factor (EGF), basic fibroblast growth factor (bFGF), or transforming growth factor (TGF) and others produced by e.g., fibroblasts or smooth muscle cells. Thus, the cultivation and positive adherence of cell clusters consisting of those cell types and the respective assessment and comparison, as shown here, may be helpful for considering a mesh regarding its possible tissue ingrowth and capacity of formation of connective tissue. Coating of meshes with plasma and ATR seem to have a positive effect on those features.

## 5. Conclusion

Plasma coating of meshes leads to an improved biocompatibility score in a novel *in vitro* test system warranting *in vivo* assessment of the procedure.

## Figures and Tables

**Table 1:** Meshes.

Mesh	Material	Biomechanic characteristics
Vitamesh, Proxy Biomedical	Large pore monofilament Polypropylene	Knit polypropylene, pore size 2410 *µ*m, Thickness (microns) 250, tear resistance (*F*_max⁡_ N) 33.7
Dynamesh, FEG Textiltechnik	Monofilament (PVDF) polyvinylidene fluoride	Effective porosity: 58%, reactive surface: 1.97 m²/m², suture pull out strength: 31 N, tear propagation resistance: 28 N, pore size: 3000 *μ*m
TFT Motifmesh, Proxy Biomedical	Micromachined polytetrafluoroethylene	Pore size 235 *µ*m, thickness (microns) 150, tear resistance (*F*_max⁡_ N) 15.1
TVT polypropylene	Polypropylene	Nonabsorbable, permanent polypropylene suture, pore size of 164 × 96 *μ*m
UltraPro Hernia System Medium (UHSM), Ethicon	Polypropylene reinforced with poliglecaprone fibers	Filament thickness 0.09 mm, mesh thickness 0.5 mm, (*F*_max⁡_ N) 69 N, pore size 300 *µ*m
Proceed surgical mesh, Ethicon	Monofilament polypropylene encapsulated with polydioxanone (PDS)	Closely knitted with small pores <1000 *µ*m size, high tensile strength
Mersilene, Johnson and Johnson	Multifilament mesh, polyethylene terephthalate	Density 0,19 g/cm^3^, pore size 120–85 *µ*m

Investigated meshes and their main characteristics [5].

**Table 2:** Mesh ranking.

Mesh type	Native	PBMC	ATR	Plasma
Dynamesh, FEG Textiltechnik	2,2	2,1	2,6	2,7
TFT Motifmesh, Proxy Biomedical	2,0	2,0	2,3	2,7
Vitamesh, Proxy Biomedical	1,6	1,4	1,7	1,9
Ultrapro hernia system medium (UHSM), ethicon	1,4	1,4	1,6	1,6
Mersilene band, Johnson and Johnson	1,2	1,2	1,4	1,5
Proceed surgical mesh, ethicon	1,2	1,1	1,4	1,5
TVT polypropylene	1,0	1,1	1,4	1,6

For assessing the adherence score after 6 weeks, we evaluated each mesh with tissue of ten different patients. After semiquantitative determination, we conducted the frequency distribution value of the score results for each mesh. (points/10).

Scoring was based on the classification proposed by Melman et al. [10].

None (0 point): no tissue ingrowth.

Fair (1 point): thin bands of fibroblasts and small collagen deposits between mesh filaments.

Good (2 points): moderately thick bands of fibroblasts and collagen deposits between mesh filaments.

Excellent (3 points): nearly all spaces between mesh filaments occupied by fibroblasts, collagen deposits, and capillaries.

## Abbreviations

FBR: Foreign body reaction
FDA: American food and drug administration
POP: Pelvic organ prolapse
SUI: Stress urinary incontinence
EGF: Epidermal growth factor
bFGF: Basic fibroblast growth factor
TGF: Transforming growth factor
PBMC: Peripheral blood mononuclear cell
ATR: Advanced tissue regeneration system
TVT: Tension free vaginal tape
CD: Cluster of differentiation.
